# Intra-oral orthosis vs amitriptyline in chronic tension-type headache: a clinical and laser evoked potentials study

**DOI:** 10.1186/1746-160X-2-15

**Published:** 2006-05-25

**Authors:** Marina de Tommaso, Elliott Shevel, Carla Pecoraro, Michele Sardaro, Daniela Divenere, Olimpia Di fruscolo, Paolo Lamberti, Paolo Livrea

**Affiliations:** 1 Neurological and Psychiatric Sciences Department University of Bari, Bari, Italy; 2 The Headache Clinic Johannesburg, South Africa; 3 Odontostomatologic and Chirurgic Department University of Bari, Bari, Italy; 4 Clinica Neurologica II, Policlinico, Piazza Giulio Cesare 11, I-70124 Bari, Italy

## Abstract

**Background:**

In the present study, we examined clinical and laser-evoked potentials (LEP) features in two groups of chronic tension-type headache (CTTH) patients treated with two different approaches: intra-oral appliance of prosthesis, aiming to reduce muscular tenderness, and 10 mg daily amitriptyline.

**Methods:**

Eighteen patients with diagnosed CTTH participated in this open label, controlled study. A baseline evaluation was performed for clinical features, Total Tenderness Score (TTS) and a topographic analysis of LEPs obtained manually and the pericranial points stimulation in all patients vs. healthy subjects. Thereafter, patients were randomly assigned to a two-month treatment by either amitriptyline or intra-oral appliance.

**Results and discussion:**

Both the intra-oral appliance and amitriptyline significantly reduced headache frequency. The TTS was significantly reduced in the group treated with the appliance. The amplitude of P2 response elicited by stimulation of pericranial zones showed a reduction after amitriptyline treatment.

Both therapies were effective in reducing headache severity, the appliance with a prevalent action on the pericranial muscular tenderness, amitriptyline reducing the activity of the central cortical structures subtending pain elaboration

**Conclusion:**

The results of this study may suggest that in CTTH both the interventions at the peripheral and central levels improve the outcome of headache.

## Introduction

Although tension-type headache is the most common type of primary headache, its pathophysiology is poorly understood. The best documented abnormality in patients with tension type headache is increased pericranial myofascial tenderness [1,2]. Pericranial tenderness has been shown to be positively associated with both the intensity and the frequency of tension type headache [2,3]. It is generally accepted that myofascial tenderness probably plays a key role in the pathophysiology of tension type headache. Recently, a pathophysiological model for tension type headache has been proposed. Accordingly, the main problem is central sensitization at the level of the spinal dorsal horn/trigeminal nucleus, resulting from prolonged nociceptive inputs from pericranial myofascial tissues. This central sensitization is posited to cause supraspinal sensitization and central neuroplastic changes, that possibly lead to increased pericranial muscle activity [4].

In a recent study we examined features of Laser evoked potentials (LEPs) [5,6], as well as cutaneous heat-pain thresholds to laser stimulation, in relation to the tenderness of pericranial muscles in chronic tension type headache (CTTH), during a pain-free phase [7]. The amplitude of the N2-P2 complex elicited by stimulation of the pericranial zone was greater in CTTH patients than in controls; the amplitude increase was significantly associated with the Total Tenderness Score (TTS) [8]. Our findings suggested that pericranial tenderness may be a primary phenomenon that precedes headache, mediated by increased pain awareness at the cortical level.

In our previous study, we postulated that a cortical hyper-vigilance to the pericranial muscles was correlated with muscle tenderness, which may be aggravated or generated by a high level of cortical arousal [7]. The role of peripheral factors in the induction of a specific hyper-attention to painful stimuli at pericranial sites, and the efficacy of a specific intervention at peripheral level in improving the outcome of headache is presently unclear. Peripheral nociception at the level of pericranial muscles may be reduced by specific interventions aiming to reduce the muscular tenderness. Previously, intra-oral orthoses, designed to act on the bite, have been shown to be effective in the treatment of myofascial pain and headache pain originating in the pericranial muscles [9,10]. In this study we used an intra-oral non-occluding appliance, originally designed for the treatment of myofascial pain and headache related to muscle tension [11].

Amitriptyline is the only established prophylactic treatment of CTTH [12,13] and it has been the drug of choice for chronic pain since 1964 [14]. Though the mode of action of amitriptyline is not fully understood, evidence suggests that it acts at the central level by inhibiting the neuronal re-uptake of norepinephrine and serotonin in the brain [12], with an effect quite independent from its antidepressant action [15].

In a previous study we have described in brief form the effects of amitryptiline and intra-oral appliance on the clinical and LEPs features of CTTH patients [16]. The aim of the present study was to describe in detail the effect of a specific intervention at the peripheral level consisting of an intra-oral appliance, compared to the central effect of amitriptyline, on the LEPs, the TTS and the main clinical features of two groups of CTTH patients.

## Methods

### Subjects

Eighteen outpatients attending the Headache Centre of the Neurology Clinic of Bari University, who fulfilled the criteria of CTTH associated with a pericranial muscles tenderness, according to International Headache Society (code 2.3.1) [17], participated in the study. All patients had been attending the practice for at least 6 months, during which they had been requested to register all headache episodes in a diary. All patients underwent a standardized interview as well as a clinical neurological, psychiatric and dental examination: they were also examined by Zung's Self-rating Anxiety Scale (SAS) [18] and Self-rating Depression Scale [19] (SDS).

The clinical features of the patients are summarized in Table 1. Subjects with general medical, neurological, psychiatric (according to American Psychiatric Association, 1994) diseases, and patients who were taking psychoactive drugs, prophylactic treatments for headache, or had displayed over-use of analgesic drugs in the last 2 months, were excluded. In addition, criteria for exclusion also included dental malocclusions, existence of any removable dental appliance, and/or acute oral conditions. All patients who participated were instructed to attend to the recording session free from pain and free of medication intake for at least the previous 12 h. Longer intervals were not possible, since most of the patients experienced daily headache (Table 1).

**Table 1:** Clinical features of chronic tension type headache patients.

Patients	Age	Sex	Age of illness (years)	Frequency of headache (days with headache/month)	Totale Tenderness score	Self-evaluating Anxiety Scale (Zung)	Self-evaluating Depression Scale (Zung)
**1**	**55**	**F**	**2**	**30**	**11**	**31**	**32**
2	35	M	3	15	3	30	32
**3**	**55**	**F**	**1**	**30**	**7**	**35**	**36**
4	34	M	3	16	3	31	33
**5**	**54**	**F**	**2**	**30**	**7**	**36**	**35**
6	34	M	3	15	2	24	25
**7**	**51**	**F**	**10**	**15**	**11**	**57**	**38**
8	48	F	1	15	3	40	36
**9**	**30**	**M**	**2**	**30**	**4**	**31**	**27**
10	34	M	2	30	3	37	33
**11**	**29**	**F**	**3**	**21**	**4**	**32**	**29**
12	33	F	2	30	3	36	42
**13**	**35**	**M**	**10**	**20**	**8**	**36**	**34**
14	49	M	1	30	5	37	33
**15**	**18**	**F**	**5**	**30**	**3**	**35**	**34**
16	46	F	20	16	7	48	39
**17**	**20**	**M**	**6**	**21**	**3**	**32**	**36**
18	45	F	12	17	7	45	46

The patients reported in outlined characters, were assigned to amitriptyiline, the remainder to the appliance appliance.

All patients gave their informed consent to the study, which was ethically approved by the of Neurological and Psychiatric Science Department of Bari University). The clinical examination and recording session were carried out between 12 and 55 hours after the end of the last headache (mean 23 ± 12.2 hours), in the basal condition. The TTS was performed by manual palpation by one neurologist with experience in headache, who was experimentally blinded to the assigned treatment. The right frontalis, masseter, temporalis, pterygoid, sternocleidomastoid, and trapezius muscles, and the sternocleidomastoid and neck muscle insertions were examined using the TTS system. This method uses a combination of behavioural and verbal items, each of which is scored on a four-point Likert scale, defined as: 0 denial of tenderness, no visible reaction; 1 verbal report of discomfort or mild pain, no visible reaction; 2 verbal report of moderate pain, with or without visible reaction; 3 verbal report of marked pain and visible expression of discomfort, according to Langermark and Olesen [20]. The LEP recording was performed at least 1 h after the TTS examination.

### CO_2 _laser stimulation and LEP recording

Each subject was seated in a comfortable chair positioned in a quiet room with an ambient temperature of 21–23 °C, in an awake and relaxed state, with eyes closed. Subjects and experimenters wore protective goggles during data acquisition. The pain stimulus was a laser pulse (wavelength 10.6 μm) generated by a CO_2 _laser (Neurolas, Electronic Engineering, Florence, Italy; ). The beam diameter was 2.5 mm and the duration of the stimulus pulse was 20 ms. Signals were recorded through 19 disk electrodes, according to the 10–20 International System (impedance below 5000 ohms), referring to the nasion with the ground at Fpz. Another electrode was placed above the right eye to record the electrooculogram (EOG). Signals were amplified, filtered (0.5–80 Hz), and stored on a biopotential analyser (Micromed System Plus; Micromed, Mogliano Veneto, Italy; ). Time analysis was for 1s, at a sampling rate of 512 Hz. Trials contaminated by ocular or muscle artefacts were excluded from the analysis. An automatic artefact rejection system excluded from the average all runs containing transient signals exceeding 65 mV on any recording channel, including the EOG.

### Stimulation

Cutaneous heat stimuli were delivered to the dorsum of the right hand (RH), and to the skin above the right frontalis, masseter, temporalis, sternocleidomastoid, and trapezius muscles, and to the neck muscle insertions. The site of stimulation was visualized by a laser beam. The location of the impact on the skin was adjusted slightly between two successive stimuli to avoid the sensitization of the nociceptors and nociceptor fatigue. A 7.5-W laser intensity with 25 ms duration was used in each case [21]. Subjects were requested to report the quality of sensation (pain rating :PR) after each stimulus presentation using a visual analogue scale (VAS) in which 0 indicated no pain in white, increasing in a gradual scale of reds to 100, which indicated severe pain. Two series of 20 stimuli were delivered in each case with an interstimulus interval of 10 s. The order of the stimulation sites was modified randomly across patients and controls.

### LEPs analysis

LEP recordings were analyzed by an investigator blind to the clinical conditions. Blocks of at least 15 trials free from artefacts were averaged offline. A grand average across the two series of stimuli was obtained for each patient. LEPs were identified based on their latency and distribution, and three responses, N1, N2 and P2, were labelled [22]. Absolute latencies of scalp potentials were measured at the highest peak of each response component and the amplitude of each wave was measured from the baseline. The N1 component was measured on the temporal derivation (T3), the P2 and N2 components were analysed at the vertex (CZ). In addition, each case was evaluated for the amplitude of N1 referring off-line the contralateral temporo-parietal derivations to Fz reference : the mean amplitude values across T5-Fz;T3-Fz;P3-Fz was the temporal N1. We also computed the amplitude of N2 (vertex N2) and P2 (vertex P2), computing in each case the mean value across the vertex and midline derivations (Cz-Fz-Pz, C3, C4, referred to the nasion).

### Experimental procedure

**Figure 1:**
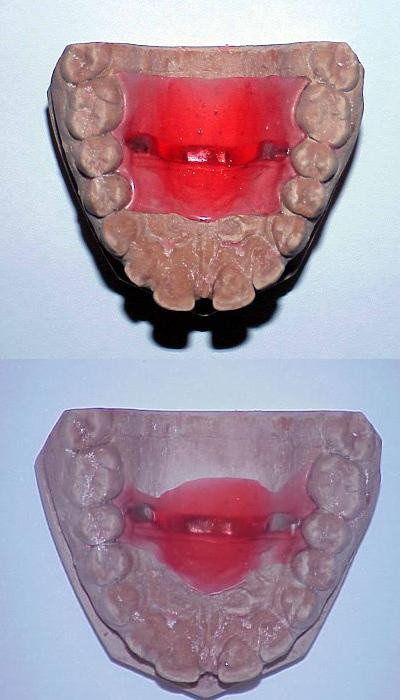
An example of oral appliance before (upper) and after (lower) adjustement.

A basal evaluation of clinical features and TTS were performed in all patients. (T0:basal conditions); patients were then consecutively allocated to the two groups: one patient was allocated to the amitriptyline group for each one who entered the intra-oral appliance group (Table 1). It was an open-label randomised study. Patients assigned to the drug therapy assumed a dosage of 10 mg amitriptyline each evening. We used a low dosage of drug, in order to obtain a therapeutic effect avoiding sedation. In the other group, an appliance designed to reduce the pericranial tenderness, was fitted. The appliance was fabricated at "The Headache Clinic" in Johannesburg, on the basis of their previous experience of its efficacy in treating primary headache (Fig.1)[11]. The appliance was constructed on the plaster casts of the selected patients' maxillary teeth: the devices were fitted to each patient, and adjusted in order to not interfere with normal speech. The final shape and thickness of the prostheses was different for each patient [16], depending on the shape of the tongue and palate, and the tongue movements during speech. Patients in this group were instructed to wear the appliance night and day for two consecutive months. Patients in the amitriptyline group were instructed to take the drug every evening for two consecutive months, during which they should observe any side-effect. We joined all patients weekly by telephonic interview, to test their compliance. After two months (T1), they were requested to come again free from pain and analgesic treatment for at least 12 hours. The mean frequency of headache in the last two months was computed and the TTS and LEPs were performed, according to the methods described above. The TTS and LEP evaluations were done at least 12 hours after removal of the appliance or the last amitriptyline intake. In addition, patients were submitted again to Zung's Self-rating Anxiety Scale (SAS) [18] and Self-rating Depression Scale (SDS) [19].

### Statistic analysis

The comparison between the two treated groups was performed by the univariate ANOVA, using the conditions T0 and T1 and the type of treatment as factors and the frequency of headache, the TTS, the PR, the temporal N1 and the vertex N2 and P2 as variables.

## Results

### Clinical features

**Figure 2:**
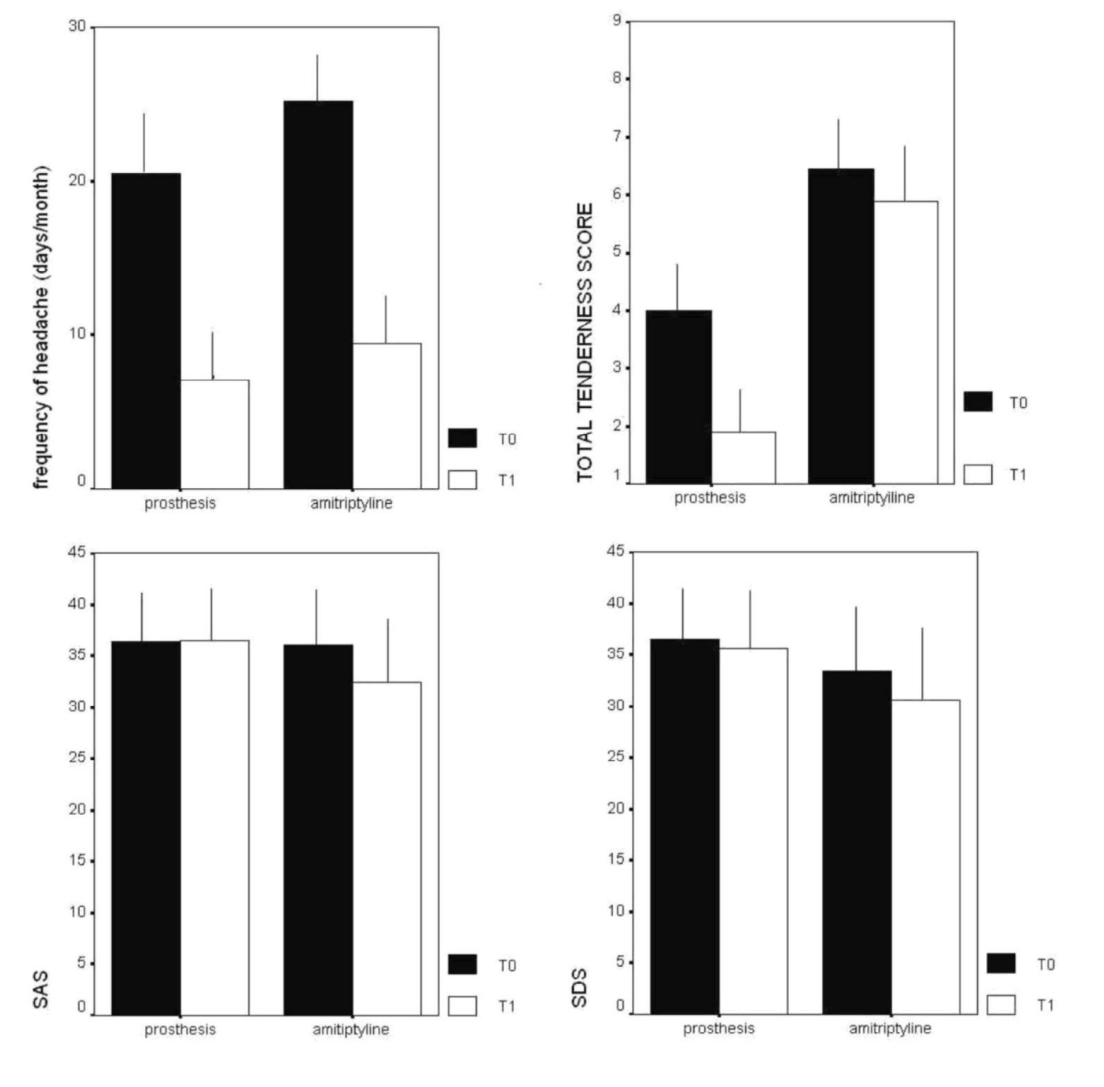
**Clinical Features of chronic tension type headache patients**. Mean values and standard deviations of the main clinical features, frequency of headache, Total Tenderness Score (TTS), Self Evaluating Anxiety Scale (SAS) and Self Evaluating Depression Scale (SDS) in chronic tension type headache in basal conditions (T0) and after two months treatment (T1) by appliance (n°9 patients) and amitriptyline (n°9 patients).

No patient reported significant side effects with the appliance: one of them experienced slight speech disturbance after a month, and the appliance had to be adjusted further. Two patients reported drowsiness due to the amitriptyline, but this did not interfere with their daily activities, and, therefore, continued with the medication regimen. All the clinical features were similar in the two selected groups and ANOVA was employed for treatment as a factor. Both the oral appliance and amitriptyline significantly reduced headache frequency (Fig.2): the two-way ANOVA with the treatment and the condition as factors, showed a significant effect of the treatment (F = 56.5 p < 0.0001), which was not dissimilar between the two groups (treatment × condition : F = 0.4 p = 0.53). The TTS was significantly different between the two groups, for reduced values in the group treated by the appliance (ANOVA with treatment as factor F = 17.11 p = 0.0001): any way, the difference between the two groups approached the statistical significance when the effect of the condition was considered (ANOVA with condition × treatment as factors: F: 4.36 p = 0.052). (Fig.2) The anxiety levels, tested by the Zung scale, were similar in the two groups (ANOVA with treatment as factor: F = 0.86 p = 0.35), the effect of the condition was not statistically relevant (condition × treatment: F = 0.64;p = 0.42). The depression levels were reduced in the group treated by amitriptyline, but the effect of condition × treatment did not reach statistic significance (condition × treatment : 3.86 p = 0.066). (Fig. 2).

The pain rating of laser stimulus in the two treatments was not significantly different at any of the stimulated sites.

### LEPs

**Figure 3:**
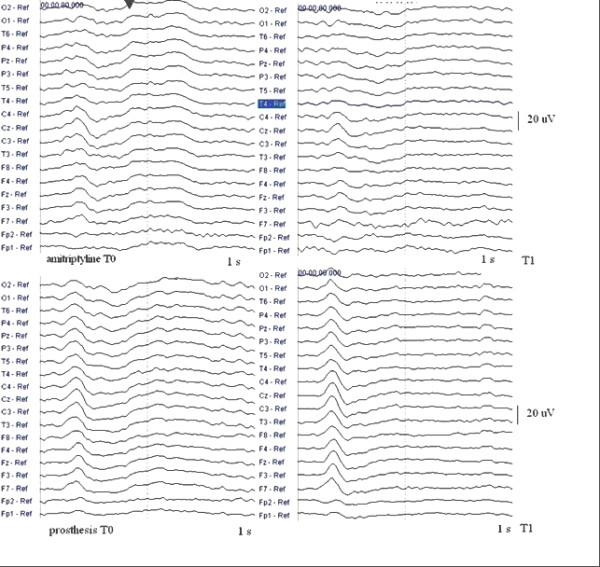
**Laser evoked potentials in chronic tension type headache patients**. Grand average of LEPs across 9 chronic tension type headache patients assigned to amitriptyline and 9 assigned to the oral appliance in basal conditions (T0) and after two months treatment (T1). The amitriptyline induced a reduction of the later positive wave, the P2.

The amitriptyline provoked a reduction of the vertex P2 (Table 2) at the neck point, masseter, and temporal sites (Fig 3). The oral device did not reduce the amplitude of LEPs at any stimulated point (Table 2). The mean percent rate of reduction of the vertex P2 across the whole range of pericranial sites was 35.4 ± 12.6 in amitriptyline group, and 9.9 ± 7.8 in the appliance treated group (ANOVA with cases as factor: F = 25.4 p < 0.001). In the amitriptyline group, it was correlated with the percentage rate of reduction of headache frequency (Pearson correlation test: 0.71; p 0.049), but it was not correlated with the mean percentage reduction of TTS, SAS and SDS.

**Table 2:** Laser evoked potential features in chronic tension type headache patients

				Neck	front.	mass	ster.	temp	trap.	hand
T0	AMYT	n1 temp	M	-1	-3	-2	0,4	-2	-2,3	-3
		uV	SD	3,2	5	2,1	3,3	2	5,3	5,3
T1			M	-1,5	-3	-3	-1,2	-3,2	-2,4	-2
			SD	2	6,2	2,2	6,6	4	5,4	4,5
T0	PROST.		M	-3	-2	2,1	-3	-3	-2,3	-2
			SD	8	8	3,2	7,1	4	6,5	3,4
T1			M	-1	-1	1,4	-3,1	-2,2	-2,1	-1,6
			SD	1,9	5,5	4,2	-3,2	3	4,5	4,5
T0	AMYT	n2 vert	M	-12	-16,2	-16,6	5,3	-16,2	-9,8	-16
		uV	SD	7	14,3	6	7	3,5	6,6	14,3
T1			M	-11	-16,3	*-8	-12,3	-8,8	-7	-16,2
			SD	10,9	11,7	4	8,9	8,4	7,5	9,8
T0	PROST.		M	-7,3	-19	12,7	-8	-13	-8,4	-12,3
			SD	10,1	15,5	4,6	12	10,2	13,4	4,5
T1			M	-15,1	-19,2	10	-12,4	-9,8	-13,5	-10,4
			SD	11,2	11,2	10,3	9,1	9,3	20,1	10,4
T0	AMYT	p2 vert	M	16,6	14,9	21	18	21,4	15,7	11,3
		uV	SD	6	10	9,2	8	9	6,7	9,9
T1			M	*11	11,2	*11,3	15,4	*13,5	12,3	14,3
			SD	6	11	6	3,4	5	8,9	10,4
T0	PROST.		M	15	10	10,8	11,3	13,4	11,2	10,3
			SD	9	6	2	5	10,3	5,5	2,4
T1			M	11	11	10,2	11,4	14,5	9,9	10,5
			SD	11,1	14	11,3	12	14,3	7,2	2,6

Mean values (M) and standard deviations (SD) of laser evoked potentials (LEPs) amplitude in chronic tension type headache amytriptiline group (n°9) and oral appliance group (n°9), computed in basal conditions (T0) and after 2 months treatment (T1). Results of ANOVA with condition as factor and LEPs amplitudes as variable within the amytriptiline group. * p < 0.05

## Discussion

A significant weakness of our study design was the lack of placebo. This was unavoidable due to the fact that we were unable to design a "placebo" intra-oral appliance. Instead we compared two different therapeutic approaches, one with a prevalent effect at the peripheral and the other at the central level. This method may be limited, because an oral device may produce a placebo effect, which acts at the central level producing an analgesic effect. A recent review stated that the gold standard of pharmacological research, the double blind placebo control trial, cannot readily be emulated in other forms of therapeutic design, as the behavioral therapy trials [23]: in the case of the appliance, the design of an intra-oral applicance without any effect on muscular tenderness, may be supposed for further evaluations. The appliance was efficient in reducing the frequency of headache. Its action is probably a result of its effect on muscle tension, as previously observed [11]. The group treated with the appliance showed a significant reduction of pericranial tenderness compared to the group treated by amitriptyline. The appliance failed to reduce LEP amplitudes, so the peripheral effect on muscle contraction seemed to be efficacious despite it did not reduce the cortical response to experimental painful pericranial stimulation. If tension type headache patients showed a tendency toward a self generation of pericranial muscular tenderness by a mechanism of cortical origin [7], action at the muscular level may improve headache: the pericranial tenderness generates phenomena of sensitization at peripheral and central levels, which cause the persistence and the extension of headache, limited by an intervention at muscular level. The oral appliance was well tolerated in our series and no patient dropped out because of serious adverse effects. As in previous studies [13], amitriptyline was efficacious in reducing headache, and was well tolerated in all patients. Although statistical analysis did not show any relevant difference between the two treatments, the effect of amitriptyline on headache frequency appeared superior to that of the appliance, despite the low dosage and the scarce effect on the pericranial tenderness. Though the mode of action of amitriptyline is not fully known, evidence has shown that it acts at the central level by inhibiting the neuronal re-uptake of norepinephrine and serotonin in the brain [12], with an effect quite independent from its antidepressant action. As previously reported, [15] depression and anxiety were not reduced in our patients by amitriptyline, in relation to clinical efficacy on headache. Amitriptyline reduced the amplitude of the vertex LEP complex when most of the pericranial points were stimulated, confirming its inhibiting effect at the level of the pain-modulating cortex. This reducing effect on LEP amplitude was not linked with the decline of TTS, which remained high during the headache-free phase of the amitriptyline-treated group. In previous studies [7,24] we suggested that TTS is mediated by a cortical hyper-attention to pericranial points and that the muscular pain should be centrally mediated. In this study we have observed that amytriptiline reduced the cortical activation by pericranial painful laser stimuli leaving quite unmodified the muscular tenderness, accordingly the appliance reduced TTS without reducing the LEPs. The tendency toward a self-generation of pericranial pain was not completely reversed in chronic tension type headache patients by a therapeutic approach at the peripheral and central levels, which appeared efficient on headache. Amitriptyline seemed to act on the central pain modulating system, specially on the nociceptive cortex which generates the late P2 wave. The reduced activity of the cortical zones devoted to the emotive and attentive components of pain [24], improves the suffering of tension-type headache, even if the pericranial tenderness persists. On the other hand the reduction of pericranial tenderness induced by the appliance alleviates headache, despite the persistence of a high level of cortical activation against pericranial painful stimuli.

## Conclusion

The pericranial tenderness, whose generation, according to our studies, is favoured by cortical hyper-attention to pericranial sites [7,14], may initiate a self sustaining circuit in which prolonged nociceptive input from pericranial myofascial tissues causes central sensitization at the level of the spinal dorsal horn/trigeminal nucleus, with supraspinal sensitisation and further activation of cortical nociceptive areas, which increase the pericranial muscle activity and the painful afferent stimuli. [4]

The results obtained within the experimental conditions of the present study indicate that intervention at both the peripheral and central levels may interrupt this reverberating circuit, improving the outcome of headache.

## Competing interests

'The author(s) declare that they have no competing interests.

## Authors' contributions

M D carried out conception and design, analysis and interpretation of data

MS and CP carried out acquisition of evoked potentials and clinical data.

E S carried out the appliance design and acquisition of clinical data.

D D carried out acquisition of clinical data.

P L and P Li. approved the final version to be submitted
